# Effectiveness of HEPA Filters at Removing Infectious SARS-CoV-2 from the Air

**DOI:** 10.1128/msphere.00086-22

**Published:** 2022-08-10

**Authors:** Hiroshi Ueki, Michiko Ujie, Yosuke Komori, Tatsuo Kato, Masaki Imai, Yoshihiro Kawaoka

**Affiliations:** a Division of Virology, Institute of Medical Science, University of Tokyo, Tokyo, Japan; b Center for Global Viral Diseases, National Center for Global Health and Medicine, Tokyo, Japan; c Shinwa Corporation, Tokyo, Japan; d Department of Special Pathogens, International Research Center for Infectious Diseases, Institute of Medical Science, University of Tokyo, Tokyo, Japan; e Department of Pathobiological Sciences, School of Veterinary Medicine, University of Wisconsin—Madison, Madison, Wisconsin, USA; Mount Sinai School of Medicine

**Keywords:** COVID-19, HEPA filter, SARS-CoV-2, aerosols, air cleaner

## Abstract

Coronavirus disease 2019 (COVID-19) spreads by airborne transmission; therefore, the development and functional evaluation of air-cleaning technologies are essential for infection control. Air filtration using high-efficiency particulate air (HEPA) filters may be effective; however, no quantitative assessment of the effectiveness of these filters in the removal of infectious severe acute respiratory syndrome coronavirus 2 (SARS-CoV-2) from the air has been reported. To evaluate the removal effect of HEPA filtration on airborne SARS-CoV-2, here, we disseminated infectious SARS-CoV-2 aerosols in a test chamber in a biosafety level 3 facility and filtered the air with a HEPA-filtered air cleaner in the chamber. The air cleaner with the HEPA filter continuously removed the infectious SARS-CoV-2 from the air in a running-time-dependent manner, and the virus capture ratios were 85.38%, 96.03%, and >99.97% at 1, 2, and 7.1 ventilation volumes, respectively. The air-cleaning performance of a HEPA filter coated with an antiviral agent consisting mainly of a monovalent copper compound was also evaluated, and the capture ratio was found to be comparable to that of the conventional HEPA filter. This study provides insights into the proper use and performance of HEPA-filtered air cleaners to prevent the spread of COVID-19.

**IMPORTANCE** Air filtration simulation experiments quantitatively showed that an air cleaner equipped with a HEPA filter can continuously remove SARS-CoV-2 from the air. The capture ratios for SARS-CoV-2 in the air when the air cleaner was equipped with an antiviral-agent-coated HEPA filter were comparable to those with the conventional HEPA filter, and there was little effect on SARS-CoV-2 in the air that passed through the antiviral-reagent-coated HEPA filter.

## OBSERVATION

The airborne transmission of severe acute respiratory syndrome coronavirus 2 (SARS-CoV-2) is a key infectious route for the spread of coronavirus disease 2019 (COVID-19) (1, 2). It has been suggested that air filtration using high-efficiency particulate air (HEPA) filters might be effective in reducing SARS-CoV-2 wafting in the air (3). In the United States, the guidelines and recommended practices of the Institute of Environmental Sciences and Technology (IEST-RP-CC001) define an efficient HEPA filter as one that captures more than 99.97% of submicrometer particles at 0.3 μm (4). Air filtration was found to substantially remove SARS-CoV-2 in experimental room air when viral RNA was measured by using quantitative reverse transcription-PCR (qRT-PCR) (3, 5, 6). The effects of air filtration on the removal of infectious SARS-CoV-2 particles have also been evaluated by using aerosolized bacteriophages as mimetic viruses (7); however, no reports to date have quantitatively evaluated the effectiveness of HEPA filters in the removal of infectious SARS-CoV-2 particles (size range of approximately 60 to 140 nm) contained in aerosols (size range of approximately 0.001 to 100 μm) from the air (8, 9).

**FIG 1:**
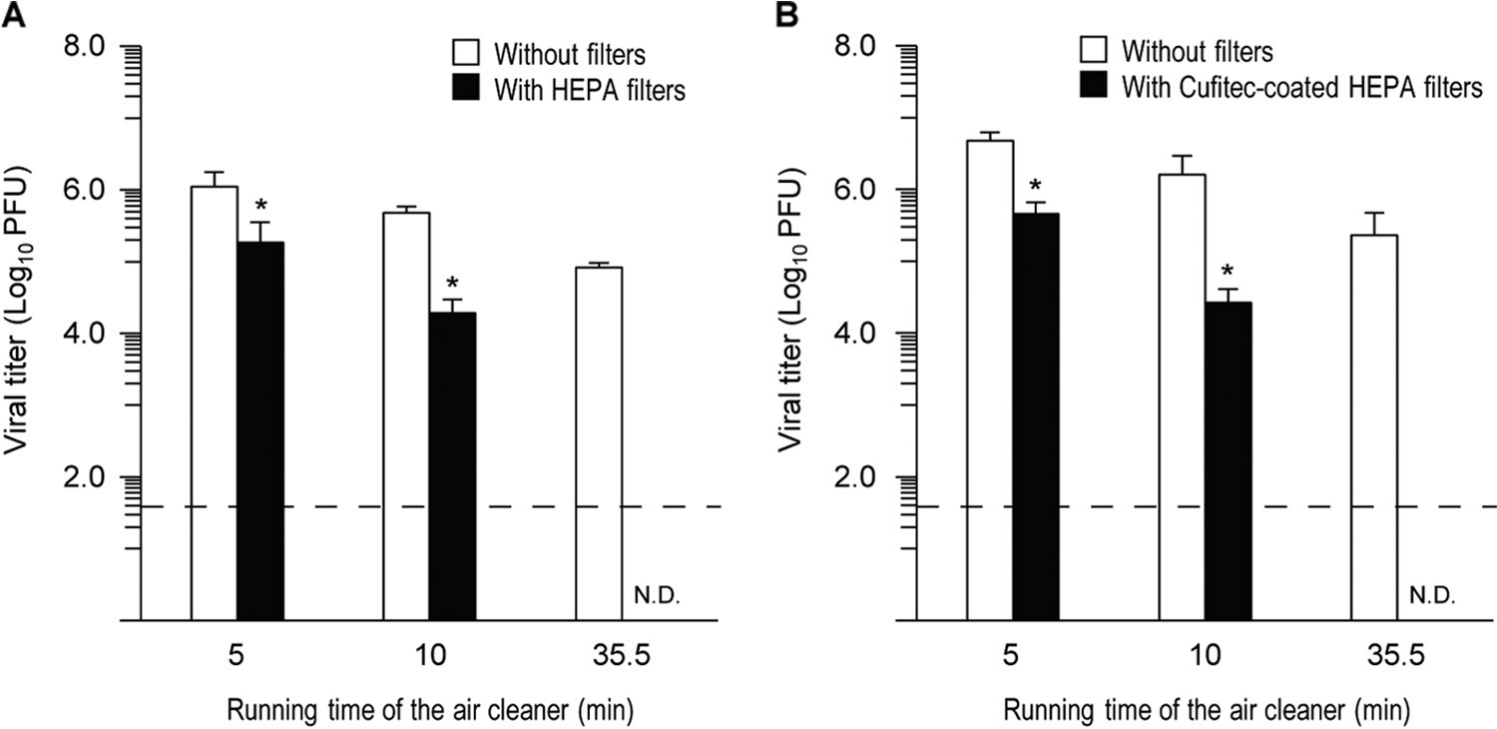
Effectiveness of an air cleaner with filters on airborne SARS-CoV-2. A test chamber (240 L) was constructed in a biosafety cabinet at a biosafety level 3 facility, and viral aerosols were generated by using a nebulizer (charged with 6 mL of a viral suspension) in the chamber. An air cleaner with a HEPA filter (A) or a Cufitec-coated HEPA filter (B) in the chamber was operated at a flow rate of 48 L/min and a face velocity of 2.1 cm/s for the indicated times. After air cleaning, the airborne SARS-CoV-2 in the chamber was collected by using an air sampler, and the infective viral loads were measured by the use of a plaque assay. Data are presented as means ± standard deviations (SD). N.D., none detected. The dashed line indicates the detection limit. The experiments were repeated three times (*n* = 3). * indicates significant differences compared to the control group (air cleaners without filters with the same running time) (*P < *0.05).

To investigate the filtration efficacy of HEPA filters, we constructed a test chamber in a biosafety level 3 (BSL3) facility and placed an air cleaner with a HEPA filter in the chamber (see Fig. S1 in the supplemental material). A compressor nebulizer connected to one side plate of the chamber was charged with 6 mL of a SARS-CoV-2 suspension to generate virus droplets/aerosols. The generated particles (initial-mass median diameter, 5.5 ± 0.2 μm) may have become smaller as they wafted in the test chamber during the experiments (10). After the chamber was filled with virus aerosols, the air cleaner in the chamber was operated for 5, 10, or 35.5 min, providing 12 chamber volume filtrations per h. After air filtration, the viral aerosols still wafting through the chamber were collected by an air sampler, and a plaque assay was used to determine the viral titer (11, 12) (Text S1). The air cleaner with the HEPA filter removed the infectious SARS-CoV-2 from the chamber in a running-time-dependent manner (Fig. 1A). The virus capture ratios with the HEPA filter were 85.38%, 96.03%, and >99.97% when the filtration times were 5, 10, and 35.5 min, respectively (Table S1).

10.1128/msphere.00086-22.1 TEXT S1 Supplemental materials and methods. Download Text S1, DOCX file, 0.04 MB. Copyright © 2022 Ueki et al. 2022 Ueki et al. https://creativecommons.org/licenses/by/4.0/ This content is distributed under the terms of the Creative Commons Attribution 4.0 International license.

10.1128/msphere.00086-22.2 FIG S1 Schematic representation of the system used to test air-cleaning performance. The test chamber was constructed in a biosafety cabinet in a biosafety level 3 facility. One side plate of the chamber was connected to a customized compressor nebulizer, and the other side was connected to an air sampler. A customized air cleaner with a HEPA filter was placed in the chamber, and the running time was controlled remotely. The nebulizer was charged with 6 mL of a virus suspension (5.5 × 10^6^ PFU/mL with 1% bovine serum albumin [BSA]) to generate aerosols. After the chamber was filled with the aerosols, the cleaner with the HEPA filter was operated for 5, 10, or 35.5 min at a flow rate of 48 L/min, and the viral aerosols still wafting through the chamber were collected. Download FIG S1, TIF file, 0.1 MB. Copyright © 2022 Ueki et al. 2022 Ueki et al. https://creativecommons.org/licenses/by/4.0/ This content is distributed under the terms of the Creative Commons Attribution 4.0 International license.

10.1128/msphere.00086-22.3 TABLE S1 Effectiveness of the air cleaner with a HEPA filter at capturing SARS-CoV-2. The virus capture ratio was evaluated by using the viral titers when the air cleaner with the HEPA filter was operated for the times indicated. Download Table S1, XLSX file, 0.01 MB. Copyright © 2022 Ueki et al. 2022 Ueki et al. https://creativecommons.org/licenses/by/4.0/ This content is distributed under the terms of the Creative Commons Attribution 4.0 International license.

We next tested the filtration efficacy of a HEPA filter coated with the antiviral reagent Cufitec. Cufitec is a monovalent copper compound that inactivates viruses such as influenza virus and feline calicivirus by generating OH radicals without hydrogen peroxide (13, 14). The efficiency of airborne SARS-CoV-2 removal by the Cufitec-coated HEPA filter was comparable to that of the regular HEPA filter, with capture ratios of 90.35%, 98.34%, and >99.99% at filtration times of 5, 10, and 35.5 min, respectively (Fig. 1B and Table S2).

10.1128/msphere.00086-22.4 TABLE S2 Effectiveness of the air cleaner with a Cufitec-coated HEPA filter at capturing SARS-CoV-2. The virus capture ratio was determined by plaque assays when the air cleaner with the Cufitec-coated HEPA filter was operated for the times indicated. Download Table S2, XLSX file, 0.01 MB. Copyright © 2022 Ueki et al. 2022 Ueki et al. https://creativecommons.org/licenses/by/4.0/ This content is distributed under the terms of the Creative Commons Attribution 4.0 International license.

Our study shows that air filtration using HEPA filters can consistently remove infectious SARS-CoV-2 from the air. Under our experimental conditions, approximately 90% of the infectious SARS-CoV-2 still wafted in the air after the filtration of 1 chamber volume, and at least 7.1 chamber volumes were required to reduce the viral load to below the detection limit. This finding indicates that the air in the chamber does not pass through the air cleaner evenly and that there are areas where the aerosols tend to linger. Therefore, when using an air cleaner, in addition to using a HEPA filter, it would be desirable to filtrate the entire room, including areas where air tends to be congested. Alternatively, an air cleaner system in combination with air ventilation may achieve more efficient air cleaning in a short time.

The capture ratios for SARS-CoV-2 in the air when the HEPA filter was coated with an antiviral agent were comparable to those with the conventional HEPA filter, and there was little effect on SARS-CoV-2 in the air that passed through the antiviral-reagent-coated HEPA filter. Although only one type of antimicrobial coating was tested in our study, and other, additional products should be evaluated, this finding suggests that once aerosols are captured on HEPA filters, they do not detach (15, 16); therefore, antiviral reagents on HEPA filters may have a slight effect on the removal of infectious SARS-CoV-2 from the air. Several methods of applying antiviral treatments to air filters have been attempted, and they appear to have inactivation effects on pathogens on the filter surface (17–19); therefore, applying antiviral reagents to HEPA filters may reduce the risk to personnel who change filters.

Our data provide valuable information on the proper use and performance of HEPA-filtered air purifiers in hospitals and in daily life and will help in determining whether they need to be used in combination with other protective equipment (e.g., face masks or room ventilation) to prevent the spread of COVID-19.
