# Supplementary material for: Effectiveness of HEPA Filters at Removing Infectious SARS-CoV-2 from the Air
Source: mSphere. 2022 Aug 10;7(4):e00086-22. doi: 10.1128/msphere.00086-22 (PMC9429918; doi:10.1128/msphere.00086-22)
Supplement: TEXT S1 [file msphere.00086-22-s0001.docx]

**Supplementary information**

**Materials**

*Virus*

SARS-CoV-2/UT-NCGM02/Human/2020/Tokyo was propagated in VeroE6/TMPRSS2 cells with Dulbecco’s modified Eagle’s medium (DMEM) as described previously^1-3^. VeroE6/TMPRSS2 (JCRB1819) cells^4^ were obtained from the National Institutes of Biomedical Innovation, Health and Nutrition, Japan.

*Filters*

The high-efficiency particulate arrestance (HEPA) filters and Cufitec-coated HEPA filters were provided by Shinwa Corporation.

**Methods**

*Air cleaning performance test of an air cleaner with HEPA filters*

To investigate the cleaning effect of an air cleaner with HEPA filters, a test chamber (1200-mm long x 400-mm wide x 500-mm high) was constructed in a biosafety cabinet at our biosafety level 3 facility (Supplementary Figure 1). A jet-type nebulizer (NE-C28, Omron) was connected to one side plate of the chamber, and charged with 6 ml of virus suspension [5.5 × 10^6^ plaque forming units (PFU)/ml with 1% of bovine serum albumin (BSA)] to generate aerosols. The particles sprayed by the nebulizer (initial mass median diameter, 5.5 ± 0.2 μm) may become smaller as they wafted in the test chamber during experiments^5^. After the chamber was filled with virus aerosols, a customized air cleaner (filtration type) with HEPA filters within the chamber was operated for 5, 10, or 35.5 minutes at a flow rate of 48 L/min and a face velocity of 2.1 cm/s. The customized air purifier was a miniaturized version of an air purifier typically used in a hospital room for patients with infectious diseases modified to fit the volume of the test chamber, with an applied volume and airflow rate approximately 1/417 of those of a hospital room. The face velocity on the HEPA filter and the ventilation frequency (12 ACH) of the customized air purifier were set to simulate those an air purifier in a hospital room. Then, the viral aerosols still wafting through the chamber were collected by a gelatin membrane (#12602-080-ALK; diameter, 8.0 cm; pore size, 3.0 µm; SARTORIUS) set in an air sampler (MD8 Airscan: filter-type, SARTORIUS). The membrane in which the viral particles were trapped was immediately dissolved in 10 ml of DMEM containing 5% fetal calf serum (FCS) and the viral titer was assessed by use of a plaque assay as described previously^3,6^.

*Virus titration assay*

Confluent VeroE6/TMPRSS2 cells in 6-well plates were infected with 200 μl of a dilution of the virus suspension. After incubation for 1 h at 37 °C, the virus inoculum was removed and the cells were overlaid with 1% agarose solution in DMEM with 5% FCS. The plates were incubated for 48 h and then the agar-covered monolayers were fixed with 10% neutral buffered formalin. The plaques were counted after removal of the agar. The capture ratio of the HEPA filters was calculated by using the following formula: (viral titer without filters - viral titer with filters) / (viral titer without filters) x100.

*Statistical analysis*

Data are presented as the mean ± SD. Student's *t* test was performed and differences were considered to be statistically significant when the *p*-value was < 0.05.

**Acknowledgements**

We thank S. Watson for editing the manuscript. We also thank K. Iwatsuki-Horimoto, M. Ito, and M. Okuda (University of Tokyo) for technical assistance. This research was supported by a Research Program on Emerging and Re-emerging Infectious Diseases from the Japan Agency for Medical Research and Development (AMED) (JP19fk0108113, JP19HA1003), by a Japan Program for Infectious Diseases Research and Infrastructure from AMED (JP21wm0125002), and by the NIAID-funded Center for Research on Influenza Pathogenesis (CRIP; HHSN272201400008C).

**Author Contributions**

H.U., M.U., Y. Komori., T.K., M.I., and Y. Kawaoka. designed and performed the experiments. H.U. and Y. Kawaoka. wrote the manuscript.

**Competing financial interests**

Yoshihiro Kawaoka has ongoing unrelated collaborations and/or sponsored research agreements with Daiichi Sankyo Pharmaceutical, Toyama Chemical, Tauns Laboratories, Inc., Shionogi & Co. LTD, Otsuka Pharmaceutical, and KM Biologics and has received royalties from MedImmune and Integrated Biotherapeutics. Yousuke Komori and Tatsuo Kato are employed by the Shinwa Corporation, which holds patents “JP6378551” and “JP2020-203242”.

**Supplemental references**

1 Halfmann, P. J. *et al.* Transmission of SARS-CoV-2 in Domestic Cats. *The New England journal of medicine*, doi:10.1056/NEJMc2013400 (2020).

2 Imai, M. *et al.* Syrian hamsters as a small animal model for SARS-CoV-2 infection and countermeasure development. *Proceedings of the National Academy of Sciences* **117**, 16587-16595, doi:10.1073/pnas.2009799117 (2020).

3 Ueki, H. *et al.* Effectiveness of Face Masks in Preventing Airborne Transmission of SARS-CoV-2. *mSphere* **5**, doi:10.1128/mSphere.00637-20 (2020).

4 Matsuyama, S. *et al.* Enhanced isolation of SARS-CoV-2 by TMPRSS2-expressing cells. *Proc Natl Acad Sci U S A* **117**, 7001-7003, doi:10.1073/pnas.2002589117 (2020).

5 Berg, E. B. & Picard, R. J. In vitro delivery of budesonide from 30 jet nebulizer/compressor combinations using infant and child breathing patterns. *Respir Care* **54**, 1671-1678 (2009).

6 Hatagishi, E. *et al.* Establishment and clinical applications of a portable system for capturing influenza viruses released through coughing. *PloS one* **9**, e103560, doi:10.1371/journal.pone.0103560 (2014).
