# Supplementary figures and images for: Effectiveness of HEPA Filters at Removing Infectious SARS-CoV-2 from the Air
Source: mSphere. 2022 Aug 10;7(4):e00086-22. doi: 10.1128/msphere.00086-22 (PMC9429918; doi:10.1128/msphere.00086-22)

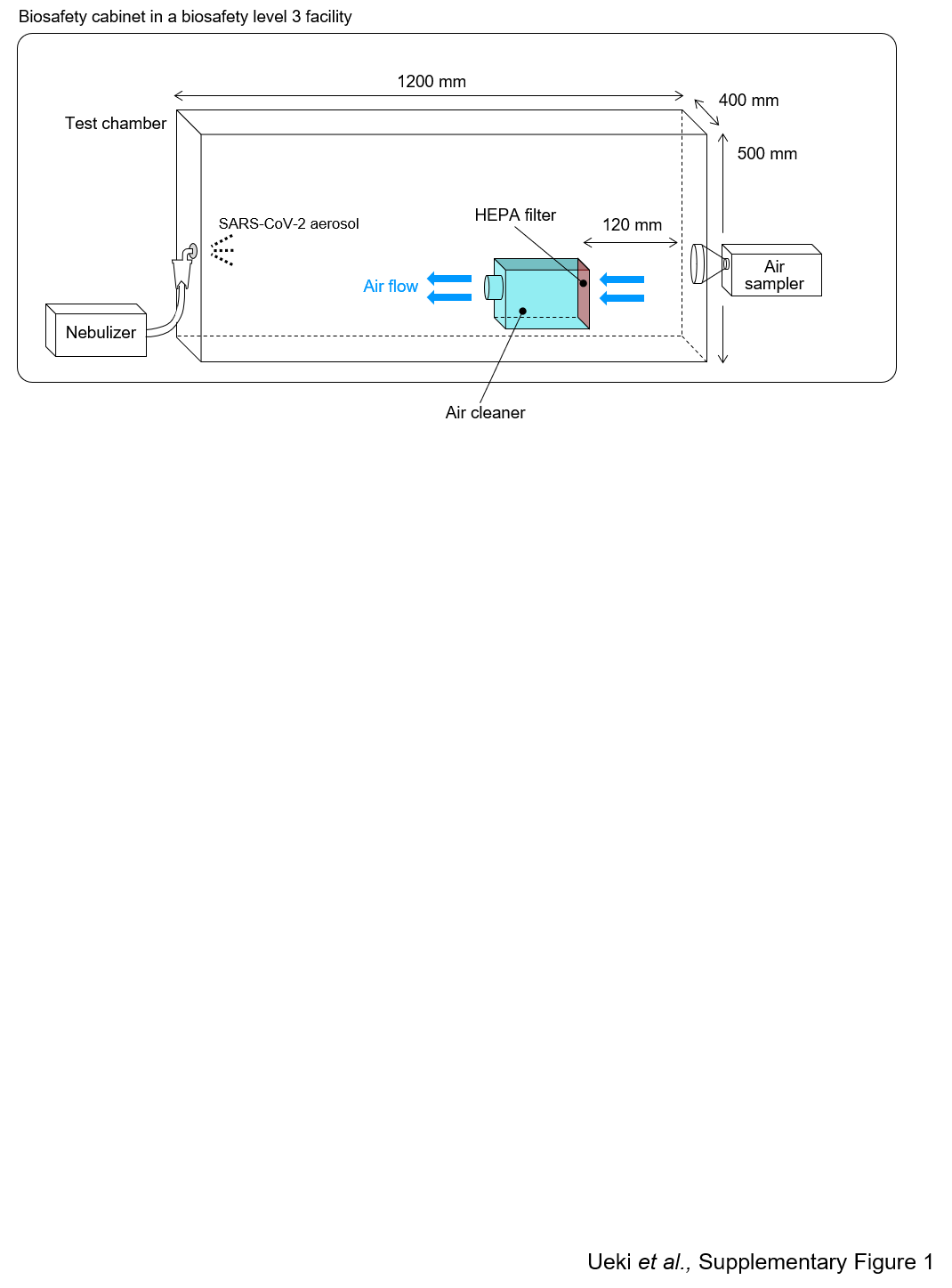

Supplement: FIG S1 [file msphere.00086-22-s0002.tif]
